# Association of periodontitis with reduced kidney function and albuminuria in early chronic kidney disease: a population-based study

**DOI:** 10.1038/s41368-026-00435-6

**Published:** 2026-04-06

**Authors:** Christian Schmidt-Lauber, Merle Ebinghaus, Katrin Borof, Berit Lieske, Alexandre Klopp, Christina Thompson, Loujain Wees, Zambaka Dawood, Guido Heydecke, Thomas Beikler, Tobias B. Huber, Ghazal Aarabi

**Affiliations:** 1 III. Department of Medicine, University Medical Center Hamburg-Eppendorf, Hamburg, Germany; 2 Hamburg Center for Kidney Health (HCKH), University Medical Center Hamburg-Eppendorf, Hamburg, Germany; 3 Department of Periodontics, Preventive and Restorative Dentistry, University Medical Center Hamburg-Eppendorf, Hamburg, Germany; 4 Department of Prosthetic Dentistry, Center for Dental and Oral Medicine, University Medical Center Hamburg-Eppendorf, Hamburg, Germany

**Keywords:** Outcomes research, Biomarkers, Epidemiology

## Abstract

Periodontitis has been linked to chronic kidney disease (CKD) through systemic inflammation. However, evidence in early CKD remains limited. We analyzed 6 179 participants from a population-based cohort (median age 62 years; 51% women). Periodontitis was classified according to the 2017 American Academy of Periodontology / European Federation of Periodontology criteria. Kidney function was assessed by the combined creatinine- and cystatin C–based estimated glomerular filtration rate (eGFR) and urinary albumin-to-creatinine ratio (uACR). Associations of periodontitis stages and mean clinical attachment loss (CAL) with eGFR and uACR were examined using multivariable linear regression adjusted for age, sex, diabetes, and smoking. Mediation analyses tested indirect effects of high-sensitivity C-reactive protein (hsCRP) and interleukin-6 (IL-6). Prevalence of severe periodontitis increased from 14% in individuals with normal kidney function (eGFR ≥60 mL/min per 1.73 m²) to 36% in those with moderately reduced eGFR (<60 mL/min per 1.73 m²) and from 21% in individuals without albuminuria (<10 mg/g) to 32% in those with moderately increased albuminuria (29–300 mg/g). After adjustment, Stage IV periodontitis was independently associated with lower eGFR (β−1.08 mL/min per 1.73 m²; 95% CI−2.04 to −0.12) and higher Blom-transformed uACR (β 0.09; 95% CI 0.01–0.16) compared with Stage I/II. hsCRP partially mediated these associations, accounting for 35% of the association with eGFR and 10% with uACR. These findings suggest that both inflammatory and non-inflammatory pathways may link periodontitis to early CKD. Periodontitis was associated with reduced eGFR and higher uACR in early CKD. While overlapping risk factors contribute, an independent association remained, only partly explained by systemic inflammation.

## Introduction

Periodontitis, a chronic inflammatory disease affecting the tooth-supporting structures, is one of the most prevalent inflammatory conditions worldwide, affecting up to 50% of the global population.^1–3^ Its association with cardiovascular diseases has been extensively documented, with systemic inflammation emerging as a key mediator in this relationship.^4,5^

Periodontitis also exhibits a high prevalence among individuals with advanced or end-stage chronic kidney disease (CKD).^6,7^ CKD itself is also a highly prevalent condition, affecting approximately 10% of the global population.^8^ It often remains undiagnosed and is associated with considerable morbidity and mortality.^9,10^ While risk factors such as arterial hypertension and diabetes are well-established contributors to CKD progression,^11^ emerging evidence suggests that periodontal disease may also constitute an important but underrecognized modifiable risk factor in CKD.^7,12–17^ Consistent associations between periodontitis and CKD have been demonstrated in multiple observational studies, with more severe forms of periodontitis correlating with greater impairments in kidney function.^17,18^

Shared risk factors, including diabetes, low physical activity, and adverse social determinants, undoubtedly contribute to the association between the two disease entities.^12^ Both conditions are further closely related to increasing age and its associated low-grade inflammation state,^19^ characterized by immune changes such as impaired neutrophil function, which contribute to a sustained inflammatory state in the oral cavity or systemic circulation.^20^ This chronic, low-grade inflammation can both worsen periodontal tissue destruction and contribute to CKD progression.^19^ In addition to inflammation, periodontitis and CKD also share other key pathophysiological mechanisms, including endothelial dysfunction and oxidative stress.^21,22^ Thus, the association between periodontitis and CKD appears to be bidirectional.^23^

Several findings indicate that this relationship may not only be due to shared exposures but also extend to an independent association. Persistent subclinical inflammation, a hallmark of CKD pathophysiology, may be sustained and amplified by periodontitis.^24,25^ Periodontitis involves chronic changes in the composition of the oral microbiome, triggering persistent immune activation.^26^ Systemic inflammation resulting from such changes in the oral microbiome is thought to play a central role.^27–29^ This is supported by studies demonstrating systemic inflammation and vascular dysfunction in individuals with periodontitis,^30,31^ as well as experimental data showing direct endothelial invasion by periodontal pathogens.^32^ Oral microbes and inflammatory mediators (e.g., high-sensitivity C-reactive protein (hsCRP), interleukin-6 (IL-6)) enter the systemic bloodstream through inflamed gingival tissues and reach distant organs, potentially triggering remote site inflammation.^27,29^ This systemic vascular manifestation of periodontitis could contribute to extraoral organ damage, including glomerular injury and endothelial dysfunction in the kidneys.^33–35^

Conversely, CKD itself is also characterized by systemic inflammation and immune dysregulation, which increase susceptibility to infections and may thereby directly exacerbate periodontitis.^36,37^ In addition, recent small observational studies suggest that the oral microbiome is altered in patients with advanced CKD, possibly facilitating the evolution or progression of periodontitis.^29,38–40^

However, despite extensive research, prior studies remain heterogeneous regarding the independent association between periodontitis, systemic inflammation, and kidney health. Evidence on the early stages of CKD is particularly limited.^17^ Establishing this relationship could be crucial for identifying at-risk individuals who may benefit from enhanced monitoring and preventive interventions.^7^ This underscores the need for large population-based investigations to clarify the relationship between the two disease entities at early stages, as well as the role of systemic inflammation using sensitive biomarkers in this interplay.^7,41–43^ Also, only a few prior studies have quantified albuminuria or measured cystatin C, even though inflammatory kidney damage may be more accurately reflected by changes in these parameters than by serum creatinine alone.^44,45^

Therefore, this study aimed to investigate in a large population-based cohort (P: population) whether periodontitis (E: exposure), compared to no periodontitis (C: comparison), is associated with decreased kidney function, measured by estimated glomerular filtration rate (eGFR) and urinary albumin-to-creatinine ratio (uACR) (O: outcomes). We further examined whether systemic inflammation, quantified by high-sensitive hsCRP and IL-6 levels, mediates this association. We hypothesized that periodontitis is independently associated with early CKD, and that this association is partially mediated by systemic inflammation.

## Results

### Baseline characteristics

**Fig. 1:**
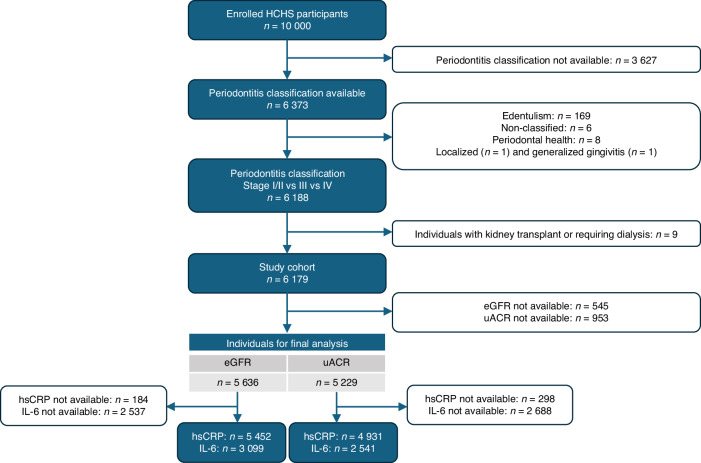
Flowchart of participant selection. Numbers are based on available data without imputation of missing variables. Sample sizes for eGFR and uACR differ due to non-overlapping missing data. eGFR estimated glomerular filtration rate, uACR urine albumin-to-creatinine ratio, hsCRP high-sensitivity C-reactive protein, IL-6 interleukin-6, HCHS Hamburg City Health Study

The study population comprised *n* = 6 179 individuals (Fig. 1) with a median age of 62 years (interquartile range (IQR) 55–69) and 51% females. Most participants had a high (46%) or medium (49%) education. Median body mass index (BMI) was 26 kg/m^2^ (IQR 23.5–29) and most prevalent reno-cardiovascular risk factors were hypertension (65%), dyslipidemia (24%), and diabetes (7.8%). hsCRP was 0.12 mg/dL (IQR 0.06–0.25) and IL-6 was 1.57 pg/mL (IQR 1.14–2.20) in median. Detailed baseline characteristics stratified by clinical periodontitis stages according to the 2017 American Academy of Periodontology (AAP)/European Federation of Periodontology (EEP) classification are shown in Table 1.^46,47^ Of the *n* = 6 179 included participants, 37% (*n* = 2 259) had Stage I/II, 42% (*n* = 2 584) Stage III and 22% (*n* = 1 336) Stage IV periodontitis. Individuals with Stage IV periodontitis were older (median age 67 vs. 59 years), more likely to be male (54% vs. 42%), and had a higher prevalence of hypertension (77% vs. 58%), dyslipidemia (31% vs. 20%), diabetes (12% vs. 5.7%) as well as a higher median BMI (26.9 kg/m^2^ vs. 25.7 kg/m^2^) compared to participants with Stage I/II periodontitis. Further, systemic inflammatory markers hsCRP and IL-6 were higher in individuals with Stage IV as compared to those with Stage I/II periodontitis (0.14 vs. 0.10 mg/dL and 1.81 vs. 1.46 pg/mL, respectively). Accordingly, both inflammatory markers and traditional cardiovascular risk factors were more prevalent in individuals with more advanced periodontitis. A comparable pattern (see Supplementary Tables S1 and S2) was found for individuals with a reduced eGFR (<60 mL/min per 1.73 m^2^) and increased uACR (≥30 mg/g), who were older, more likely to be male, had a higher prevalence of hypertension and diabetes as well as higher inflammatory markers as compared to participants with a normal eGFR (≥60 mL/min per 1.73 m^2^) or uACR (<10 mg/g).

**Table 1:** Baseline characteristics

Characteristics	Overall *N* = 6 179 (100%)	Periodontitis			*P*-value
		Stage I/II *N* = 2 259 (37%)	Stage III *N* = 2 584 (42%)	Stage IV *N* = 1 336 (22%)	
**Sociodemographics**					
Sex					<0.001
Male	3 043 (49%)	940 (42%)	1 375 (53%)	728 (54%)	
Female	3 136 (51%)	1 319 (58%)	1 209 (47%)	608 (46%)	
Age (years)	62.0 (55.0, 69.0)	59.0 (53.0, 67.0)	62.0 (55.0, 69.0)	67.0 (61.0, 72.0)	<0.001
Education					<0.001
Low	265 (4.5%)	70 (3.2%)	83 (3.3%)	112 (9.0%)	
Medium	2 906 (49%)	1 012 (47%)	1 176 (47%)	718 (58%)	
High	2 716 (46%)	1 079 (50%)	1 225 (49%)	412 (33%)	
(Missing)	292	98	100	94	
BMI (kg/m^2^)	26.0 (23.5, 29.0)	25.7 (23.3, 28.6)	25.8 (23.5, 28.9)	26.9 (24.2, 30.0)	<0.001
(Missing)	318	125	123	70	
**Risk factors**					
Smoking					<0.001
Never	2 272 (37%)	950 (42%)	987 (38%)	335 (25%)	
Current	1 126 (18%)	353 (16%)	432 (17%)	341 (26%)	
Former	2 747 (45%)	947 (42%)	1 149 (45%)	651 (49%)	
(Missing)	34	9	16	9	
Alcohol (g/d)	9.6 (2.7, 23.1)	9.7 (2.8, 21.9)	11.0 (3.6, 26.4)	6.7 (1.6, 19.4)	<0.001
(Missing)	559	187	186	186	
Diabetes	446 (7.8%)	122 (5.7%)	176 (7.5%)	148 (12%)	<0.001
(Missing)	460	128	242	90	
Hypertension	3 823 (65%)	1 248 (58%)	1 580 (65%)	995 (77%)	<0.001
(Missing)	273	89	141	43	
Dyslipidemia	1 370 (24%)	423 (20%)	558 (24%)	389 (31%)	<0.001
(Missing)	406	111	212	83	
Coronary Artery Disease	229 (4.0%)	64 (3.0%)	93 (3.8%)	72 (6.1%)	<0.001
(Missing)	425	130	148	147	
**Inflammatory Biomarker**					
IL-6 (pg/mL)	1.57 (1.14, 2.20)	1.46 (1.04, 2.05)	1.53 (1.12, 2.14)	1.81 (1.40, 2.68)	<0.001
(Missing)	2 999	1 064	1 242	693	
hsCRP (mg/dL)	0.12 (0.06, 0.25)	0.10 (0.06, 0.22)	0.11 (0.06, 0.24)	0.14 (0.08, 0.33)	<0.001
(Missing)	372	138	167	67	
**Kidney function**					
uACR (mg/g)	4.2 (2.5, 8.1)	4.0 (2.5,7.2)	4.2 (2.4, 8.0)	4.8 (2.6, 10.0)	<0.001
(Missing)	950	372	397	181	
eGFR (mL/min per 1.73 m^2^)	81.5 (71.9, 91.2)	83.7 (73.8, 92.9)	82.0 (72.5, 91.4)	77.2 (67.2, 86.6)	<0.001
(Missing)	543	205	210	128	
**Dental parameters**					
Sites/mouth CAL ≥ 3 mm	36.5 (19.8, 57.4)	18.5 (9.5, 30.8)	45.2 (29.8, 61.0)	58.3 (39.7, 77.3)	<0.001
Mean CAL	2.4 (2.0, 2.8)	2.0 (1.7, 2.3)	2.6 (2.3, 3.0)	2.9 (2.5, 3.6)	<0.001
Cumulative CAL > 3 mm	61.0 (21.0, 135.0)	16.0 (4.0, 35.0)	105.0 (54.0, 184.0)	112.0 (58.5, 205.0)	<0.001
Sites/mouth PD ≥ 4 mm	2.4 (0.0, 8.0)	0.0 (0.0, 1.3)	4.2 (1.3,9.6)	8.3 (2.7, 18.7)	<0.001
Mean PD	2.1 (1.8, 2.4)	1.9 (1.6, 2.1)	2.2 (2.0, 2.5)	2.4 (2.0,2.8)	<0.001
Plaque Index	8.0 (0.0, 27.8)	3.1 (0.0, 15.8)	11.1 (0.0, 30.8)	16.7 (0.0, 50.0)	<0.001
(Missing)	85	20	40	25	
DMFT-Index	19.0 (15.0, 23.0)	18.0 (15.0, 21.0)	18.0 (15.0, 21.0)	24.0 (21.0, 28.0)	<0.001
BOP Index	7.7 (1.9, 20.4)	3.8 (0.0, 10.7)	10.7 (3.7, 25.0)	13.2 (4.3, 31.7)	<0.001
(Missing)	97	57	28	12	
PISA	81.8 (18.3, 232.9)	34.9 (0.0, 101.9)	135.5 (40.2, 338.7)	111.3 (27.9, 271.9)	<0.001
(Missing)	97	57	28	12	
Number of missing teeth	2.0 (1.0, 6.0)	2.0 (0.0, 4.0)	1.0 (0.0, 3.0)	9.0 (6.0, 14.0)	<0.001

Periodontitis was classified according to the 2017 AAP/EFP periodontitis classification.^46,47^ Numbers are median (IQR) for continuous and *n* (%) for categorical parameters. Pearson’s chi-squared or Kruskal–Wallis rank-sum test was used for comparison between clinical periodontitis categories. *AAP* American Academy of Periodontology, *BMI* body mass index, *BOP* bleeding on probing, *CAL* clinical attachment loss, *EEP* European Federation of Periodontology, *eGFR* estimated glomerular filtration rate, *hsCRP* high-sensitivity C-reactive protein, *IL-6* interleukin-6, *PD* probing depth, *PISA* periodontal inflamed surface area, *ACR* urinary albumin-to-creatinine ratio

### Periodontitis and dental parameters across clinical CKD categories based on eGFR

The distribution of periodontitis stages and dental parameters across clinical eGFR categories for CKD is shown in Table 2. With declining kidney function, the prevalence of advanced periodontitis increased. In individuals with normal kidney function (CKD G1, eGFR ≥ 90 mL/min per 1.73 m^2^), 43% had Stage I/II, 43% Stage III, and 14% Stage IV periodontitis, whereas in moderate CKD G3 (eGFR 59–mL/min per 1.73 m^2^), Stage I/II decreased to 23% and Stage IV increased to 36%. In individuals with severe CKD (G4/5, eGFR < mL/min per 1.73 m^2^), only 20% had Stage I/II, and 47% had Stage IV periodontitis (*p*-value for trend <0.001). The mean clinical attachment loss (CAL) also increased from a median of 2.3 (IQR 2–2.7) in individuals with normal kidney function (G1) to 2.4 (IQR 2–2.9) in CKD G2, 2.6% (IQR 2.2–3.2) in CKD G3, and 2.6 (IQR 2.3–3.9) in CKD G4/5 (p-value for trend <0.001). Similar distributions were observed for the sites/mouth CAL ≥ 3 mm, the Bleeding on Probing (BOP) index, and the periodontal inflamed surface area (PISA), although the trend for PISA did not reach statistical significance. The median number of missing teeth was 2 (IQR 0-4) in individuals with CKD G1, 2 (IQR 1–6) in CKD G2, 4 (IQR 2–9) in CKD G3, and 4 (IQR 2–14) in G4/5 (*P*-value for trend <0.001).

**Table 2:** Periodontitis and dental parameters across clinical eGFR categories of CKD

	eGFR				
Dental parameters	CKD G1: ≥ 90 mL/min per 1.73 m^2^ *N* = 1 569	CKD G2: 89–60 mL/min per 1.73 m^2^ *N* = 3 650	CKD G3: 59–30 mL/min per 1.73 m^2^ *N* = 402	CKD G4/5: < mL/min per 1.73 m^2^ *N* = 15	*P*-value
Periodontitis					<0.001
Stage I/II	678 (43%)	1 282 (35%)	91 (23%)	3 (20%)	
Stage III	675 (43%)	1 527 (42%)	167 (42%)	5 (33%)	
Stage IV	216 (14%)	841 (23%)	144 (36%)	7 (47%)	
Sites/mouth CAL ≥ 3 mm	33.3 (17.3,52.0)	37.0 (19.9,58.0)	45.2 (28.0,67.3)	45.8 (28.7,88.6)	<0.001
Mean CAL	2.3 (2.0,2.7)	2.4 (2.0,2.9)	2.6 (2.2,3.2)	2.6 (2.3,3.9)	<0.001
Cumulative CAL > 3 mm	52.0 (15.0,115.0)	63.0 (22.0,142.0)	87.5 (36.0,175.0)	95.0 (54.0,171.0)	<0.001
BOP index	7.1 (1.9,19.4)	7.7 (1.9,20.4)	9.8 (3.6,23.2)	13.7 (2.2,21.1)	0.015
(Missing)	24	62	6	1	
PISA	77.9 (18.8,230.4)	78.4 (17.1,233.6)	111.5 (26.7,254.1)	101.7 (17.8,226.5)	0.062
(Missing)	24	62	6	1	
Number of missing teeth	2.0 (0.0,4.0)	2.0 (1.0,6.0)	4.0 (2.0,9.0)	4.0 (2.0,14.0)	<0.001

Periodontitis was classified according to the 2017 AAP/EFP periodontitis classification.^46,47^ Numbers are median (IQR) for continuous and n (%) for categorical parameters. Kruskal–Wallis rank-sum test was used for comparison between clinical eGFR categories. *AAP* American Academy of Periodontology, *BOP* bleeding on probing, *CAL* clinical attachment loss, *EEP* European Federation of Periodontology, *eGFR* estimated glomerular filtration rate, *PISA* periodontal inflamed surface area

### Periodontitis and dental parameters across clinical CKD categories based on uACR

Table 3 outlines the distribution of periodontitis stages and dental parameters across clinical uACR categories for CKD. In individuals without albuminuria (CKD A0, uACR < 10 mg/g), 21% Stage IV periodontitis, increasing to 32% in mildly increased albuminuria (A1, uACR 10–29 mg/g) and 39% in severely increased albuminuria (A3, uACR ≥ 300 mg/g) (*P*-value for trend <0.001). The mean CAL rose from 2.4 mm (IQR 2–2.8) among individuals with no albuminuria (A0) to 2.5 mm (IQR 2.1–3) in A1, 2.6 mm (IQR 2.2–3.2) in A2, and 2.5 mm (IQR 2.2–3) in A3 albuminuria (*P*-value for trend <0.001). This was paralleled by an increase in sites/mouth CAL ≥ 3 mm (*P*-value for trend <0.001). BOP and PISA followed a parallel trajectory but did not reach statistical significance. The number of missing teeth increased from a median of 2 (IQR 1–5) in A0 to 5 (IQR 1–10) in A3 (*P*-value for trend <0.001).

**Table 3:** Periodontitis and dental parameters across clinical uACR categories of CKD

	uACR				
Dental parameters	A0: < 10 mg/g *N* = 4 194	A1: < 30 mg/g *N* = 732	A2: < 300 mg/g *N* = 281	A3: ≥ 300 mg/g *N* = 36	*P*-value
Periodontitis					<0.001
Stage I/II	1 562 (37%)	236 (32%)	78 (28%)	11 (31%)	
Stage III	1 760 (42%)	303 (42%)	113 (40%)	11 (31%)	
Stage IV	862 (21%)	190 (26%)	89 (32%)	14 (39%)	
(Missing)	10	3	1	0	
Sites/mouth CAL ≥ 3 mm	36.1 (19.4, 56.8)	40.5 (22.3, 61.4)	46.7 (27.2, 64.7)	44.2 (21.5, 65.0)	<0.001
Mean CAL	2.4 (2.0, 2.8)	2.5 (2.1, 3.0)	2.6 (2.2, 3.2)	2.5 (2.2, 3.0)	<0.001
Cumulative CAL > 3 mm	58.0 (20.0, 132.0)	69.5 (24.0, 153.5)	77.0 (38.0, 171.0)	76.5 (26.0, 143.5)	<0.001
BOP index	7.7 (2.0, 20.0)	8.9 (1.9, 22.9)	8.7 (2.0, 20.4)	13.0 (0.9, 25.6)	0.574
PISA	79.8 (19.2, 231.5)	86.1 (19.7, 242.9)	87.0 (17.1, 230.9)	111.9 (5.8, 292.0)	0.857
(Missing)	71	11	4	0	
Number of missing teeth	2.0 (1.0, 5.0)	3.0 (1.0, 7.0)	4.0 (1.0, 8.0)	5.0 (1.0, 10.0)	<0.001

Numbers are median (IQR) for continuous and n (%) for categorical parameters. Kruskal–Wallis rank-sum test was used for comparison between clinical uACR categories. *AAP* American Academy of Periodontology, BOP: bleeding on probing, *CAL* clinical attachment loss, *EEP* European Federation of Periodontology, *PISA* periodontal inflamed surface area, *uACR* urinary albumin-to-creatinine ratio

### Continuous association of periodontitis and kidney function

**Fig. 2:**
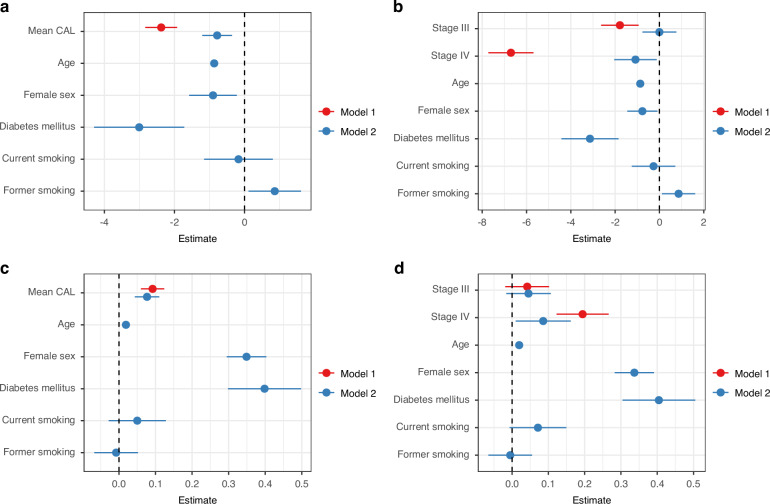
Association of eGFR and uACR with dental parameters. Multiple linear regression models investigating the association of (**a**) mean CAL with eGFR, (**b**) mean CAL with uACR, (**c**) periodontitis stages with eGFR, and (**d**) periodontitis stages with uACR. Periodontitis stages were defined according to the 2017 AAP/EFP periodontitis classification^46,47^ with Stage I/II used as the reference category. Model 1 is unadjusted, and model 2 is adjusted for age, sex, diabetes, and smoking status. Dots represent the point estimate and lines 95% CI. AAP American Academy of Periodontology, CAL clinical attachment loss, EFP European Federation of Periodontology, eGFR estimated glomerular filtration rate, uACR urine albumin-to-creatinine ratio

To better reflect the biological background and assess independent associations, we investigated the correlation of the continuous dental inflammatory parameter mean CAL as well as periodontitis stages with the numerical eGFR and uACR in multiple linear regression models (Fig. 2). After adjusting for age, sex, diabetes, and smoking status (model 2), each 1 mm increase in mean CAL was associated with a 0.78 ml/min per 1.73 m^2^ lower eGFR (95% CI: 0.36–1.21, Fig. 2a). This effect size is comparable to the decline in eGFR typically observed with one year of healthy aging.^48^ Equally, Stage IV periodontitis was independently associated with an eGFR decrease of 1.08 ml/min per 1.73 m^2^ (95% CI: 0.12–2.04,) as compared to Stage I/II periodontitis (Fig. 2b). These results showed no relevant effect modification by age, sex, diabetes, and smoking, indicating that the associations between periodontal parameters and kidney function were consistent across these subgroups (Table S3).

Similar associations were observed for periodontitis stages and mean CAL with uACR. After adjustment, the Blom-transformed uACR increased by 0.08-points (95% CI: 0.04–0.11) with every 1 mm increase in mean CAL (Fig. 2c). Similarly, Stage IV periodontitis was independently associated with a 0.09-point higher Blom-transformed uACR (95% CI: 0.01–0.16) compared to Stage I/II periodontitis (Fig. 2d). These results were generally stable across interaction analyses (Table S3). Although a nominal interaction with sex was detected for stage IV periodontitis, this did not materially affect the association between periodontal status and uACR.

### Systemic inflammation in periodontitis and CKD

**Fig. 3:**
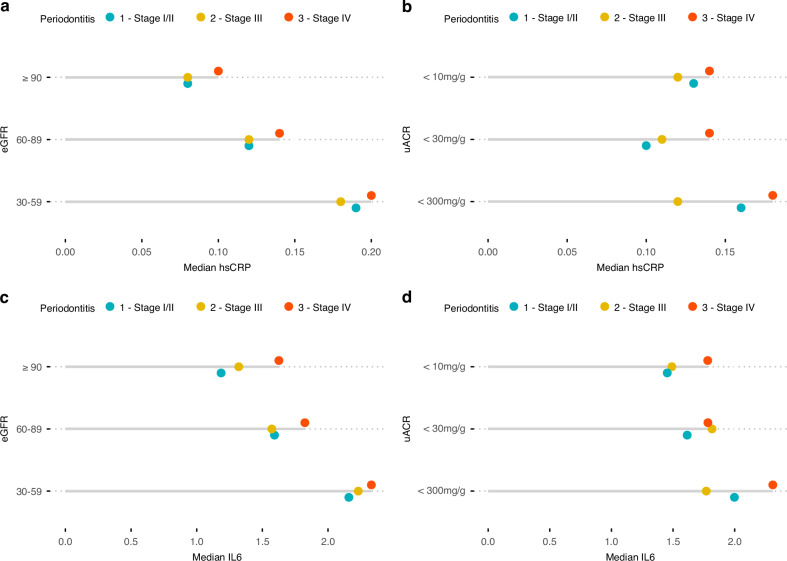
Levels of inflammatory markers across clinical CKD stages as well as periodontitis stages according to the 2017 AAP/EFP periodontitis classification^46^^,^^47^. **a** hsCRP by eGFR-based CKD stages, **b** hsCRP by uACR-based CKD stages, **c** IL-6 by eGFR-based CKD stages, and **d** IL-6 by uACR-based CKD stages. Dots represent medians of the respective inflammatory markers. AAP American Academy of Periodontology, CKD chronic kidney disease, EFP European Federation of Periodontology, eGFR estimated glomerular filtration rate, hsCRP high-sensitivity C-reactive protein, IL-6 interleukin-6, uACR urine albumin-to-creatinine ratio

To explore the underlying role of systemic inflammation in the interplay of periodontitis and CKD, we compared levels of hsCRP and IL-6 between periodontitis and CKD stages and conducted formal mediation analyses. Levels of hsCRP and IL-6 gradually increased with periodontitis stages as well as eGFR- and uACR-based CKD stages (Fig. 3a–d). The highest levels of both systemic inflammatory markers were found for individuals with the combination of the highest periodontitis and eGFR stage, as well as the highest periodontitis and uACR stage.

**Fig. 4:**
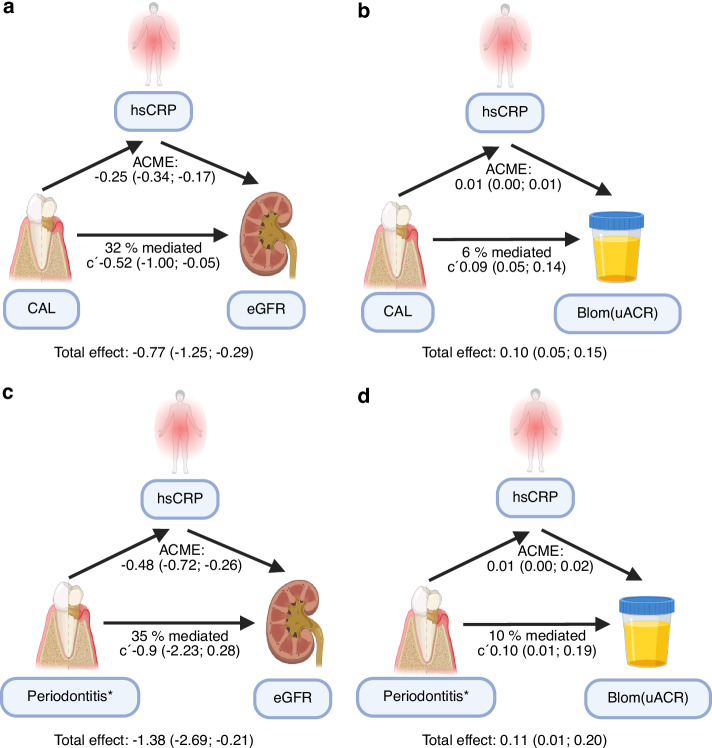
Mediation of hsCRP on the association between periodontitis and kidney function. **a** hsCRP mediation in the association between mean CAL and eGFR, **b** hsCRP mediation in the association between mean CAL and Blom-transformed uACR, **c** hsCRP mediation in the association between periodontitis stages and eGFR, and **d** hsCRP mediation in the association between periodontitis stages and Blom-transformed uACR. Periodontitis stages were defined according to the 2017 AAP/EFP periodontitis classification^46^^,^^47^, with Stage I/II used as the reference category. All models were adjusted for age, sex, diabetes, and smoking status. *For periodontitis stages, Stage IV was compared with Stage I/II. AAP American Academy of Periodontology, ACME average causal mediation effect, CAL clinical attachment loss, EFP European Federation of Periodontology, eGFR estimated glomerular filtration rate, hsCRP high-sensitivity C-reactive protein, uACR urine albumin-to-creatinine ratio. Created in BioRender. Schmidt-Lauber, C. (2026) https://BioRender.com/a18u8io

Causal mediation analyses adjusted for age, sex, diabetes, and smoking status, revealed a significant total association of the mean CAL with eGFR (β -0.774, 95% CI: −1.289–−0.273, *P* < 0.001) and uACR (β −0.100, 95% CI: 0.055–0.143, *P* < 0.001, Fig. 4a and 4b). The direct association without mediation was also significant for the association to eGFR (β −0.523, 95% CI: −1.030–−0.030, *P* = 0.032) as well as uACR (β 0.095, 95% CI: 0.050–0.137, *P* < 0.001). The indirect association mediated by hsCRP accounted for 32% in the association of mean CAL to eGFR (average causal mediation effect (ACME: β −0.251, 95% CI: −0.357–−0.168, *P* < 0.001) and 6% to uACR (ACME: β 0.006, 95% CI: 0.002–−0.001, *P* = 0.006). Similar observations were made for mediation analyses of IL-6 on the association between mean CAL and eGFR or uACR, respectively (Fig. S1).

Comparable findings were observed when periodontitis severity was categorized according to the clinically used stages of the 2017 AAP/EFP periodontitis classification (Fig. 4c and 4d). In causal mediation analyses comparing participants with stage IV periodontitis to those with stage I/II, the total associations with eGFR (β −1.377, 95% CI: −2.668–−0.135, *P* = 0.002) and uACR (β 0.107, 95% CI: 0.015–0.193, *P* = 0.022) remained significant after adjustment for age, sex, diabetes and smoking status. The indirect effects mediated by hsCRP accounted for 35% of the total association with eGFR (ACME: β −0.478, 95% CI: −0.704–−0.270, *P* < 0.001) and 10% of that with uACR (ACME: β 0.011, 95% CI: −0.002 – 0.022, *P* < 0.001). The direction of the direct, non-mediated association with eGFR was consistent with that observed for mean CAL but did not reach statistical significance (β −0.899, 95% CI: -2.171–−0.338, *P* = 0.152), probably reflecting limited power due to the less granular categorization of periodontitis severity into stages. The direct, non-mediated association with uACR remained significant (β = 0.096, 95% CI: 0.002–0.184, *P* = 0.044). Corresponding results were observed when IL-6 was examined as a potential mediator (Fig. S2).

Overall, mediation analyses showed that hsCRP explained only a minority of the association between periodontitis and kidney outcomes (35% for eGFR and 10% for uACR), indicating that most of the observed associations were independent of systemic inflammation as reflected by hsCRP.

## Discussion

This study, investigating the relationship between periodontitis and kidney function as well as the underlying role of systemic inflammation in a large population-based cohort, shows that periodontitis is associated with both eGFR and uACR already at early stages. The associations were relevant and remained significant after adjustment for major confounders, indicating a robust independent association between oral and kidney health. While the systemic inflammatory burden was highest for the combination of both diseases, it only partially mediated the independent association between periodontitis and CKD.

Our findings extend previous research by showing relevant periodontal disease according to the 2017 AAP/EFP periodontitis classification using the ACES (Application of the 2018 periodontal status Classification to Epidemiological Survey data) framework approach, even in early CKD stages. The prevalence of severe periodontitis more than doubled when kidney function declined to moderate CKD (eGFR <60 mL/min per 1.73 m^2^). While the association with uACR was weaker, the prevalence of severe periodontitis still increased by 50% in individuals with moderately increased albuminuria (uACR 30-299 mg/g). We also observed progressive changes in other important indicators of periodontal inflammation, including the mean CAL and sites/mouth CAL, which occurred even in moderately decreased eGFR or increased uACR stages. In line with previous findings from Dannewitz et al.,^14^ the number of missing teeth, a result of long-standing periodontitis, increased across CKD stages, and this trajectory also started in early CKD. Whereas most prior studies focused on advanced or end-stage CKD,^12–15,49^ our analysis highlights that clinically relevant periodontitis and its consequences are already evident in individuals with early kidney impairment (eGFR <60 mL/min per 1.73 m^2^ or uACR <30 mg/g).

Considering the association of periodontitis with eGFR and uACR stages, as well as the notable changes of important consequences of periodontitis in early CKD stages, our findings suggest that early identification and management of periodontitis may be relevant in patients with mild to moderate CKD (CKD G3a or A2 according to KDIGO). While untreated periodontitis can lead to tooth loss, in most cases, this is preventable.^50^ Thus, oral health assessments may warrant consideration in individuals with early and especially proteinuric CKD (CKD G3a or A1) and could potentially mitigate ongoing inflammation and tooth loss. Conversely, our findings underscore that assessing kidney function is important in individuals with periodontitis. Our focus on early disease stages is particularly relevant for screening strategies, which have the greatest benefit in populations where early and often undiagnosed conditions are common. However, the aim of this study was to examine the association between periodontitis and early CKD. While the findings provide important insights that may inform future screening approaches, evaluating such strategies was beyond the scope of this analysis. Therefore, recommendations for screening or intervention should be made with caution and require evidence of clinical benefit and cost-effectiveness.

When comparing levels of hsCRP and IL-6 between periodontitis and CKD stages, the inflammatory load increased with both diseases. Higher levels of systemic inflammation have been demonstrated for both conditions in previous studies as well.^51,52^ In our analyses, the highest levels of hsCRP and IL-6 were found in individuals with the combination of both the highest periodontitis as well as highest CKD stages. This highlights the increased burden of systemic inflammation when both diseases occur simultaneously, which is clinically relevant since systemic inflammation is a known driver of complications in CKD. Importantly, various studies have explicitly highlighted the role of IL-6 and its downstream molecule hsCRP in CKD progression and cardiovascular outcomes in CKD patients.^53–55^ Few studies have already investigated the effect of periodontal treatment in CKD. These studies mostly focused on small cohorts with advanced or end-stage CKD, but findings indicate that periodontal treatment can reduce systemic inflammation.^56–58^ Although a recent observational study by Wangerin et al. did not observe a correlation between periodontitis and CKD progression,^41^ other retrospective analyses demonstrated that non-surgical treatment of periodontitis was associated with a 40% lower risk of end-stage kidney disease or a 20% lower risk of hospitalization for cardiovascular disease, supporting our observations.^56^ Several factors may contribute to these differing findings. The cohort studied by Wangerin et al. consisted of younger and overall healthier individuals, potentially limiting the ability to detect associations with disease progression. In addition, periodontal status was assessed without a full-mouth examination, which may have led to underestimation of disease severity. One potential reason for these differences lies in the differences of the investigated cohorts and periodontal measurements, as the study by Wangerin et al. investigated younger and healthier individuals and did not conduct a periodontal assessment of the full mouth. Taken together, these methodological and population-based differences may partly explain the discrepant results and underscore the need for future longitudinal studies with comprehensive periodontal assessment, adequate follow-up, and a focus on individuals with early, particularly proteinuric, CKD.

While a significant part of the association between periodontitis and CKD was dependent on overlapping risk factors, we still observed a robust independent association between periodontitis and early CKD. Although the independent effect sizes were modest, they remained consistent across our analyses and may still have clinical relevance, particularly when considered at the population level. In contrast to current concepts, systemic inflammation only partially mediated this association. In formal mediation analyses, hsCRP only mediated 35% of the association between periodontitis and eGFR and 10% of the association with uACR. These results suggest that a substantial proportion of the connection between these disease entities is independent of commonly used markers for systemic inflammation. Several non-inflammatory mechanisms may contribute to this association. These include translocation of oral microbiota or their products into the systemic circulation, increased oxidative stress, shared endothelial dysfunction, and complex interactions with established metabolic risk factors such as diabetes, all of which have been implicated in both periodontal and kidney disease in prior mechanistic studies.^7,23,28,32,59,60^

The weaker impact of systemic inflammation on the association of periodontitis with uACR as compared to eGFR may reflect differences in the underlying pathophysiology of these kidney markers. While eGFR in our study was estimated using both creatinine and cystatin C, a marker known to correlate with systemic inflammation, albuminuria is considered a more direct marker of glomerular endothelial injury and altered intraglomerular hemodynamics. Thus, the pathways linking periodontitis to albuminuria may differ from those contributing to the eGFR association and may be less dependent on systemic inflammatory processes.^61,62^ However, the cross-sectional design of our study precludes causal inference, highlighting the need for future studies specifically designed to investigate causal and mechanistic links. Longitudinal studies are needed to confirm the temporal sequence between periodontitis and kidney function decline. In addition, experimental studies should explore mechanisms beyond systemic inflammation, including whether oral bacteria or their metabolites directly affect renal tissue or glomerular endothelial function, and how shared metabolic risk factors, such as diabetes, modify these associations. Finally, based on our findings, randomized controlled trials could focus on individuals with early, particularly proteinuric, CKD and moderate-to-severe periodontitis to evaluate whether non-surgical periodontal treatment influences the progression of kidney disease.

Our study has several strengths, including the investigation of a large cohort with early and mostly undiagnosed diseases. This population is well-suited to investigate associations between chronic diseases and inform the design of interventional studies to test screening and interventional strategies. Additional strengths include the evaluation of the mediating role of systemic inflammation using novel biomarkers, the application of the 2017 AAP/EFP periodontitis classification, and the detailed phenotyping of CKD, including measurements of cystatin C and uACR.

Despite its strengths, this study has several limitations. First, its cross-sectional design restricts all analyses, including the mediation approach, to testing associations rather than causal pathways. Longitudinal studies are needed to elucidate temporal dynamics and causal effects, including the role of systemic inflammation. Second, our study does not include a clinical translational analysis or assess treatment strategies. However, while not directly translational, our findings may help inform future screening approaches and research on targeted interventions. Third, while the study adjusted for a range of potential confounders, residual confounding by unmeasured variables cannot be excluded. Fourth, the dental examination in the Hamburg City Health Study (HCHS) does not specify the exact cause of tooth loss, which may be due to periodontitis - the most common cause for tooth loss^63^ - but also to other factors such as caries or trauma. Finally, the study population comprised middle-aged and older adults from a single urban area, with a potential selection bias, limiting the generalizability of the findings to other populations and age groups.

In conclusion, this study found a strong association between periodontitis and both eGFR and uACR in early stages. While overlapping risk factors play an important role, we also found a robust independent association. This association was only partially mediated by systemic inflammation, suggesting additional pathophysiological mechanisms in the interplay between periodontitis and CKD. These findings may help inform the investigation of screening strategies and further research into the molecular mechanisms underlying the oral–kidney crosstalk.

## Methods

### Study design and participants

This cross-sectional analysis is nested within the ongoing Hamburg City Health Study (HCHS), a prospective and population-based initiative in Hamburg, Germany, with the aim to identify risk factors for major chronic diseases. The rationale and design of the study have been published previously.^64^ Briefly, the HCHS includes a random sample of city residents, aged 45 to 74 years, recruited from the official inhabitant data file. All participants give written informed consent before study inclusion. The study has received approval from the local ethics committee (PV5131) and is indexed at www.ClinicalTrials.gov (NCT03934957). The participant selection for the current analysis is shown in Fig. 1. We included all participants with a complete periodontal examination among the first 10 000 HCHS participants (*n* = 6 373). According to the HCHS protocol, participants requiring endocarditis prophylaxis are not invited for dental examination. We excluded *n* = 185 individuals because a periodontitis stage according to the 2017 American Academy of Periodontology (AAP) / European Federation of Periodontology (EEP) classification could not be assigned (*n* = 169 with edentulism, *n* = 6 non-classified, *n* = 8 periodontally healthy individuals, *n* = 1 with localized, and *n* = 1 with generalized gingivitis). Also, we excluded recipients of a kidney transplant and individuals on chronic dialysis (*n* = 9), leading to a study cohort of 6 179 individuals. Of these, 5636 had available data for the estimated glomerular filtration rate (eGFR) and 5 229 for the urinary albumin-to-creatinine ratio (uACR). As missing values for eGFR and uACR did not overlap, and data are based on available cases without imputation of missing data, sample sizes differed between analyses. For analyses of inflammatory biomarkers, data from *n* = 5 452 for high-sensitive C-reactive protein (hsCRP) and *n* = 3 099 for interleukin-6 (IL-6) were available in joint analysis with eGFR as well as *n* = 4 931 for hsCRP and *n* = 2 541 for IL-6 in joint analysis with uACR (Fig. 1). All participants were investigated between 2016 and 2018.

### Data collection

All participants underwent an extensive examination comprising assessment of vital status, demographics, medical questionnaires, laboratory analyses, and dental examination.^64^ The level of education was categorized according to the International Standard Classification of Education (ISCED)-97.^65^ Smoking status was self-reported and categorized as never, former (cessation at least six months prior to participation), or current (active smoking within the last six months). Alcohol consumption (g/d) was estimated based on self-reported intake, frequency, and portion size of alcoholic beverages using the validated Food Frequency Questionnaire (FFQ), originally developed for the European Prospective Investigation into Cancer and Nutrition (EPIC) study.^66^ For hypertension, diabetes, and dyslipidemia, composite definitions were used. Diabetes was defined as a fasting blood glucose >126 mg/dL, a non-fasting blood glucose >200 mg/dL, intake of antidiabetic medication or a self-reported diagnosis. A blood pressure ≥140/90 mmHg or use of antihypertensive medication was used to define hypertension. Dyslipidemia was defined as a low-to-high-density lipoprotein (LDL/HDL) ratio >3.5 or treatment with lipid-lowering drugs. Definitions of other risk factors were based on patient interviews. Laboratory tests for blood glucose, creatinine, cystatin C, hsCRP, IL-6, blood lipids, as well as urine albumin and creatinine were performed as described previously.^67–69^ IL-6 was only measured in 5 000 individuals among the first 10 000 HCHS participants.

### Assessment of kidney function

A detailed description of the assessment of kidney function in the HCHS is provided elsewhere.^70^ Briefly, for the primary analysis, we investigated the eGFR calculated with the 2012 Chronic Kidney Disease Epidemiology Collaboration (CKD-Epi) formula for the combination of creatinine and cystatin C as well as the uACR.^71^ Staging of eGFR and uACR was performed according to the Kidney Disease: Improving Global Outcomes (KDIGO) criteria for CKD.^72^ Due to low numbers of advanced CKD, the analysis was restricted to the stages: normal eGFR (G1, ≥90 mL/min per 1.73 m^2^), mildly reduced eGFR (G2, 89–60 mL/min per 1.73 m^2^), moderately reduced eGFR (G3, 59–30 mL/min per 1.73 m^2^), and mildly reduced eGFR (combination of G4/5, <30 mL/min per 1.73 m^2^). For the uACR we added a category of slightly increased albuminuria (10–29 mg/g) to the KDIGO classification as this has previously been shown to be associated with relevant extra-renal pathologies,^73,74^ and analyzed the following uACR categories: no albuminuria (A0, <10 mg/g), mildly increased albuminuria (A1, 10–29 mg/g), moderately increased albuminuria (A2, 30–299 mg/g), and severely increased albuminuria (A3, ≥300 mg/g).

### Assessment of periodontal inflammation

All participants underwent a comprehensive oral and periodontal examination. Assessment of probing depth (PD, in mm) and gingival recession (in mm) was performed by trained and certified study nurses, using a standard periodontal probe (PCP 15, Hu-Friedy, Chicago, IL, USA) at six sites per tooth (mesio-buccal, buccal, disto-buccal, disto-palatinal, palatinal, and mesio-palatinal).

For this study, we used two main parameters of periodontitis. First, periodontitis staging was determined according to the 2017 AAP/EFP periodontitis classification^46,47^ and the ACES framework to investigate clinically used disease stages.^75^ Accordingly, study participants were categorized into edentulism, periodontal health, gingivitis, non-classified, and periodontitis. Periodontitis cases were subsequently staged into Stage I, Stage II, Stage III, and Stage IV, based on the severity of clinical attachment loss (CAL).^75^ Due to the small number of participants with Stage I periodontitis (*n* = 68), Stage I and II were combined into a single Stage I/II category. Second, we investigated the mean CAL as a continuous measure of periodontal disease. CAL is a well-established marker for the deterioration of the periodontal tissue and, in contrast to clinically used staging systems, allows for a continuous measure, which better reflects the biological nature of the disease.^46^ CAL was calculated for each tooth by adding the probing depth and the gingival recession.

Secondary dental parameters investigated in this study included the cumulative CAL (as the sum of all CAL values > 3 mm), the Bleeding on Probing (BOP) index (“Yes”/”No”) reflecting acute gingival inflammation, the PISA (mm^2^), and the number of missing teeth reflecting the result of longstanding periodontal inflammation.^76^ The BOP index was determined by probing two sites per tooth (vestibular and oral) and expressed as a percentage of bleeding sites. The PISA was calculated with a custom-made R function, based on a pre-existing and freely available Excel spreadsheet (https://www.parsprototo.info/). The formula was adapted for the two sites per tooth protocol used for BOP in the HCHS.

### Statistical analysis

Descriptive statistics are presented as medians and interquartile ranges (IQR) for continuous variables or numbers and percentages for categorical variables. Descriptive analyses were based on all participants with available data (available-case analysis). The comparison of periodontitis stages and dental parameters across eGFR and uACR categories was analyzed with the Kruskal-Wallis rank-sum test.

To better reflect biological relationships and assess independent associations, we further examined the association of clinical periodontitis stages and mean CAL with the kidney function markers eGFR and uACR through multiple linear regression models, reported as estimates with 95% CIs. Regression models were adjusted for potential confounders, including age, sex, diabetes, and smoking status. These covariates were selected based on established causal relevance for both periodontitis and CKD, supported by previous literature.^41,77,78^ Variables with less consistent or indirect evidence for causal relevance to both conditions were not included in the primary models, in order to avoid overadjustment, collinearity, and loss of statistical power, particularly when using clinically defined periodontitis stages. For periodontitis stages, Stage IV was compared to the reference category Stage I/II. To account for its skewed distribution, uACR was Blom-transformed prior to these analyses. Multicollinearity among predictors and confounders was excluded using generalized variance inflation factors (Tables S4).

For investigating the role of systemic inflammation in the association between periodontitis and CKD, levels of the systemic inflammatory parameters hsCRP and IL-6 were compared across periodontitis and CKD-stages. Additionally, we analyzed the mediating role of hsCRP and IL-6 in the association of periodontitis (Stage IV vs Stage I/II) and mean CAL with eGFR and uACR in causal mediation analyses using the *mediation* package in R.^79^ These analyses were also adjusted for potential confounders, including age, sex, diabetes, and smoking status. Multivariable regression and mediation analyses were conducted as complete-case analyses, including only participants with complete data for all variables included in the respective models.

Laboratory results below the lower limit of detection (LoD) were imputed as half the LoD. Numbers and percentages for missing values were low for nearly all variables, apart from IL-6, which was measured only in a subset of study participants (Table 1). R (version 4.4.2) was used for all statistical analyses.

## Supplementary information

Supplementary information

## Data Availability

Data will be shared upon reasonable request to the corresponding author. Data sharing is subject to approval of the HCHS steering committee.

## References

[1] Eke, P. I., Dye, B. A., Wei, L., Thornton-Evans, G. O. & Genco, R. J. & Cdc Periodontal Disease Surveillance workgroup: James Beck, G. D. R. P. Prevalence of periodontitis in adults in the United States: 2009 and 2010. *J. Dent. Res.* **91**, 914–920 (2012). 22935673 10.1177/0022034512457373

[2] Nazir, M. A. Prevalence of periodontal disease, its association with systemic diseases and prevention. *Int J. Health Sci.* **11**, 72–80 (2017). PMC5426403 28539867

[3] Chen, M. X., Zhong, Y. J., Dong, Q. Q., Wong, H. M. & Wen, Y. F. Global, regional, and national burden of severe periodontitis, 1990-2019: An analysis of the Global Burden of Disease Study 2019. *J. Clin. Periodontol.* **48**, 1165–1188 (2021). 34101223 10.1111/jcpe.13506

[4] Buhlin, K., Gustafsson, A., Pockley, A. G., Frostegard, J. & Klinge, B. Risk factors for cardiovascular disease in patients with periodontitis. *Eur. Heart J.* **24**, 2099–2107 (2003). 14643270 10.1016/j.ehj.2003.09.016

[5] Sanz, M. et al. Periodontitis and Cardiovascular Diseases. Consensus Report. *Glob. Heart* **15**, 1 (2020). 32489774 10.5334/gh.400 PMC7218770

[6] Chambrone, L. et al. Periodontitis and chronic kidney disease: a systematic review of the association of diseases and the effect of periodontal treatment on estimated glomerular filtration rate. *J. Clin. Periodontol.* **40**, 443–456 (2013). 23432795 10.1111/jcpe.12067

[7] Parsegian, K., Randall, D., Curtis, M. & Ioannidou, E. Association between periodontitis and chronic kidney disease. *Periodontol 2000* **89**, 114–124 (2022). 35244955 10.1111/prd.12431

[8] Francis, A. et al. Chronic kidney disease and the global public health agenda: an international consensus. *Nat. Rev. Nephrol.* **20**, 473–485 (2024). 38570631 10.1038/s41581-024-00820-6

[9] Tangri, N. et al. Prevalence of undiagnosed stage 3 chronic kidney disease in France, Germany, Italy, Japan and the USA: results from the multinational observational REVEAL-CKD study. *BMJ Open* **13**, e067386 (2023). 37217263 10.1136/bmjopen-2022-067386 PMC10230905

[10] Bikbov, B., Purcell, C. A., Levey, A. S., Smith, M. & Abdoli, A. GBD Chronic Kidney Disease Collaboration. Global, regional, and national burden of chronic kidney disease, 1990-2017: a systematic analysis for the Global Burden of Disease Study 2017. *Lancet* **395**, 709-733 (2020). 10.1016/S0140-6736(20)30045-3 PMC7049905 32061315

[11] Webster, A. C., Nagler, E. V., Morton, R. L. & Masson, P. Chronic kidney disease. *Lancet* **389**, 1238–1252 (2017). 27887750 10.1016/S0140-6736(16)32064-5

[12] Fisher, M. A. et al. Periodontal disease and other nontraditional risk factors for CKD. *Am. J. Kidney Dis.* **51**, 45–52 (2008). 18155532 10.1053/j.ajkd.2007.09.018

[13] Kotecha, K. et al. High prevalence of periodontal disease observed in patients on hemodialysis: a call for equitable access to dental care. *Kidney Int Rep.* **7**, 2097–2100 (2022). 36090496 10.1016/j.ekir.2022.06.016 PMC9459056

[14] Dannewitz, B. et al. Status of periodontal health in German patients suffering from chronic kidney disease-Data from the GCKD study. *J. Clin. Periodontol.* **47**, 19–29 (2020). 31603565 10.1111/jcpe.13208

[15] Schutz, J. D. S. et al. Association between severe periodontitis and chronic kidney disease severity in predialytic patients: A cross-sectional study. *Oral. Dis.* **26**, 447–456 (2020). 31742816 10.1111/odi.13236

[16] He, I. et al. Demystifying the connection between periodontal disease and chronic kidney disease - An umbrella review. *J. Periodontal Res.* **58**, 874–892 (2023). 37477165 10.1111/jre.13161

[17] Deschamps-Lenhardt, S., Martin-Cabezas, R., Hannedouche, T. & Huck, O. Association between periodontitis and chronic kidney disease: Systematic review and meta-analysis. *Oral. Dis.* **25**, 385–402 (2019). 29377446 10.1111/odi.12834

[18] Kapellas, K., Singh, A., Bertotti, M., Nascimento, G. G. & Jamieson, L. M. Periodontal and chronic kidney disease association: A systematic review and meta-analysis. *Nephrology* **24**, 202–212 (2019). 29359889 10.1111/nep.13225

[19] Clark, D., Radaic, A. & Kapila, Y. Cellular Mechanisms of Inflammaging and Periodontal Disease. *Front. Dent. Med*. 10.3389/fdmed.2022.844865 (2022). 36540609 10.3389/fdmed.2022.844865 PMC9762858

[20] Clark, D., Kotronia, E. & Ramsay, S. E. Frailty, aging, and periodontal disease: Basic biologic considerations. *Periodontol. 2000* **87**, 143–156 (2021). 34463998 10.1111/prd.12380 PMC8771712

[21] Li, S. et al. Periodontal disease and chronic kidney disease: mechanistic insights and novel therapeutic perspectives. *Front. Cell Infect. Microbiol* **15**, 1611097 (2025). 40636262 10.3389/fcimb.2025.1611097 PMC12237910

[22] Sharma, P. et al. Oxidative stress links periodontal inflammation and renal function. *J. Clin. Periodontol.* **48**, 357–367 (2021). 33368493 10.1111/jcpe.13414 PMC7986430

[23] Chapple, I. L. C., Hirschfeld, J., Cockwell, P., Dietrich, T. & Sharma, P. Interplay between periodontitis and chronic kidney disease. *Nat. Rev. Nephrol.* **21**, 226–240 (2025). 39658571 10.1038/s41581-024-00910-5

[24] D’Aiuto, F. et al. Systemic effects of periodontitis treatment in patients with type 2 diabetes: a 12 month, single-centre, investigator-masked, randomised trial. *Lancet Diab. Endocrinol.* **6**, 954–965 (2018). 10.1016/S2213-8587(18)30038-X 30472992

[25] Hajishengallis, G. & Chavakis, T. Local and systemic mechanisms linking periodontal disease and inflammatory comorbidities. *Nat. Rev. Immunol.* **21**, 426–440 (2021). 33510490 10.1038/s41577-020-00488-6 PMC7841384

[26] Hajishengallis, G. Periodontitis: from microbial immune subversion to systemic inflammation. *Nat. Rev. Immunol.* **15**, 30–44 (2015). 25534621 10.1038/nri3785 PMC4276050

[27] El-Shinnawi, U. & Soory, M. Associations between periodontitis and systemic inflammatory diseases: response to treatment. *Recent Pat. Endocr. Metab. Immune Drug Discov.* **7**, 169–188 (2013). 23909844 10.2174/18715303113139990040

[28] Li, L. et al. Periodontitis exacerbates and promotes the progression of chronic kidney disease through oral flora, cytokines, and oxidative stress. *Front Microbiol* **12**, 656372 (2021). 34211440 10.3389/fmicb.2021.656372 PMC8238692

[29] Araújo, M. V. et al. End stage renal disease as a modifier of the periodontal microbiome. *BMC Nephrol.* **16**, 80 (2015). 26055269 10.1186/s12882-015-0081-x PMC4460699

[30] de Oliveira, C., Watt, R. & Hamer, M. Toothbrushing, inflammation, and risk of cardiovascular disease: results from Scottish Health Survey. *BMJ* **340**, c2451 (2010). 20508025 10.1136/bmj.c2451 PMC2877809

[31] Lira-Junior, R., Figueredo, C. M., Bouskela, E. & Fischer, R. G. Severe chronic periodontitis is associated with endothelial and microvascular dysfunctions: a pilot study. *J. Periodontol.* **85**, 1648–1657 (2014). 25019176 10.1902/jop.2014.140189

[32] Dorn, B. R., Dunn, W. A. Jr. & Progulske-Fox, A. Invasion of human coronary artery cells by periodontal pathogens. *Infect. Immun.* **67**, 5792–5798 (1999). 10531230 10.1128/iai.67.11.5792-5798.1999 PMC96956

[33] Bui, F. Q. et al. Association between periodontal pathogens and systemic disease. *Biomed. J.* **42**, 27–35 (2019). 30987702 10.1016/j.bj.2018.12.001 PMC6468093

[34] Cullin, N., Azevedo Antunes, C., Straussman, R., Stein-Thoeringer, C. K. & Elinav, E. Microbiome and cancer. *Cancer Cell* **39**, 1317–1341 (2021). 34506740 10.1016/j.ccell.2021.08.006

[35] Chopra, A. et al. Exploring the presence of oral bacteria in non-oral sites of patients with cardiovascular diseases using whole metagenomic data. *Sci. Rep.* **14**, 1476 (2024). 38233502 10.1038/s41598-023-50891-x PMC10794416

[36] Ishigami, J. et al. CKD and Risk for Hospitalization With Infection: The Atherosclerosis Risk in Communities (ARIC) Study. *Am. J. Kidney Dis.* **69**, 752–761 (2017). 27884474 10.1053/j.ajkd.2016.09.018 PMC5438909

[37] Baciu, S. F., Mesaros, A. S. & Kacso, I. M. Chronic kidney disease and periodontitis interplay-a narrative review. *Int J. Environ. Res. Public Health*. 10.3390/ijerph20021298 (2023). 36674052 10.3390/ijerph20021298 PMC9859404

[38] Hu, J. et al. Location-specific oral microbiome possesses features associated with CKD. *Kidney Int Rep.* **3**, 193–204 (2018). 29340331 10.1016/j.ekir.2017.08.018 PMC5762954

[39] Guo, S. et al. Characteristics of human oral microbiome and its non-invasive diagnostic value in chronic kidney disease. *Biosci. Rep.*10.1042/bsr20210694 (2022). 35348181 10.1042/BSR20210694 PMC9093701

[40] Gan, Y. et al. Difference in oral microbiota composition between patients with stage 5 chronic kidney disease on hemodialysis and healthy controls. *Am. J. Transl. Res.* **15**, 3342–3354 (2023). 37303656 PMC10250997

[41] Wangerin, C. et al. Long-term association of periodontitis with decreased kidney function. *Am. J. Kidney Dis.* **73**, 513–524 (2019). 30704881 10.1053/j.ajkd.2018.10.013

[42] Ioannidou, E., Swede, H. & Dongari-Bagtzoglou, A. Periodontitis predicts elevated C-reactive protein levels in chronic kidney disease. *J. Dent. Res.* **90**, 1411–1415 (2011). 21940520 10.1177/0022034511423394 PMC3215758

[43] Niedzielska, I. et al. The odontogenic-related microinflammation in patients with chronic kidney disease. *Ren. Fail* **36**, 883–888 (2014). 24960621 10.3109/0886022X.2014.894764

[44] Beernink, J. M., van Mil, D., Laverman, G. D., Heerspink, H. J. L. & Gansevoort, R. T. Developments in albuminuria testing: A key biomarker for detection, prognosis and surveillance of kidney and cardiovascular disease-A practical update for clinicians. *Diab Obes. Metab.*10.1111/dom.16359 (2025). 10.1111/dom.16359 PMC12400485 40143452

[45] Singh, D., Whooley, M. A., Ix, J. H., Ali, S. & Shlipak, M. G. Association of cystatin C and estimated GFR with inflammatory biomarkers: the Heart and Soul Study. *Nephrol. Dial. Transpl.* **22**, 1087–1092 (2007). 10.1093/ndt/gfl744 PMC2770338 17210589

[46] Papapanou, P. N. et al. Periodontitis: Consensus report of workgroup 2 of the 2017 World Workshop on the Classification of Periodontal and Peri-Implant Diseases and Conditions. *J. Clin. Periodontol.* **45**, S162–s170 (2018). 29926490 10.1111/jcpe.12946

[47] Caton, J. G. et al. A new classification scheme for periodontal and peri-implant diseases and conditions - Introduction and key changes from the 1999 classification. *J. Clin. Periodontol.* **45**, S1–s8 (2018). 29926489 10.1111/jcpe.12935

[48] Astley, M. E. et al. Age- and sex-specific reference values of estimated glomerular filtration rate for European adults. *Kidney Int.* **107**, 1076–1087 (2025). 40122341 10.1016/j.kint.2025.02.025

[49] Kshirsagar, A. V. et al. Periodontal disease is associated with renal insufficiency in the Atherosclerosis Risk In Communities (ARIC) study. *Am. J. Kidney Dis.* **45**, 650–657 (2005). 15806467 10.1053/j.ajkd.2004.12.009

[50] Sanz, M. et al. Treatment of stage I-III periodontitis-The EFP S3 level clinical practice guideline. *J. Clin. Periodontol.* **47**(Suppl 22), 4–60 (2020). 32383274 10.1111/jcpe.13290 PMC7891343

[51] Petreski, T., Piko, N., Ekart, R., Hojs, R. & Bevc, S. Review on inflammation markers in chronic kidney disease. *Biomedicines*. 10.3390/biomedicines9020182 (2021). 33670423 10.3390/biomedicines9020182 PMC7917900

[52] Martínez-García, M. & Hernández-Lemus, E. Periodontal inflammation and systemic diseases: an overview. *Front Physiol.* **12**, 709438 (2021). 34776994 10.3389/fphys.2021.709438 PMC8578868

[53] Tonelli, M., Sacks, F., Pfeffer, M., Jhangri, G. S. & Curhan, G. Biomarkers of inflammation and progression of chronic kidney disease. *Kidney Int* **68**, 237–245 (2005). 15954913 10.1111/j.1523-1755.2005.00398.x

[54] Amdur, R. L. et al. Inflammation and progression of CKD: The CRIC Study. *Clin. J. Am. Soc. Nephrol.* **11**, 1546–1556 (2016). 27340285 10.2215/CJN.13121215 PMC5012490

[55] Koshino, A. et al. Interleukin-6 and cardiovascular and kidney outcomes in patients with Type 2 diabetes: new insights from CANVAS. *Diab Care* **45**, 2644–2652 (2022). 10.2337/dc22-0866 PMC9862371 36134918

[56] Delbove, T. et al. Effect of periodontal treatment on the glomerular filtration rate, reduction of inflammatory markers and mortality in patients with chronic kidney disease: A systematic review. *PLoS One* **16**, e0245619 (2021). 33481920 10.1371/journal.pone.0245619 PMC7822280

[57] Almeida, S., Figueredo, C. M., Lemos, C., Bregman, R. & Fischer, R. G. Periodontal treatment in patients with chronic kidney disease: a pilot study. *J. Periodontal Res.* **52**, 262–267 (2017). 27135778 10.1111/jre.12390

[58] Chaudhry, A. et al. Potential effects of non-surgical periodontal therapy on periodontal parameters, inflammatory markers, and kidney function indicators in chronic kidney disease patients with chronic periodontitis. *Biomedicines*. 10.3390/biomedicines10112752 (2022). 36359271 10.3390/biomedicines10112752 PMC9687126

[59] França, L. F. C. et al. Periodontitis changes renal structures by oxidative stress and lipid peroxidation. *J. Clin. Periodontol.* **44**, 568–576 (2017). 28419499 10.1111/jcpe.12729

[60] Randall, D. et al. Oral dysbiosis initiates periodontal disease in experimental kidney disease. *Nephrol. Dial. Transpl.* **40**, 1187–1202 (2025). 10.1093/ndt/gfae266 PMC12123317 39568053

[61] Okura, T. et al. Association between cystatin C and inflammation in patients with essential hypertension. *Clin. Exp. Nephrol.* **14**, 584–588 (2010). 20809110 10.1007/s10157-010-0334-8

[62] Benzing, T. & Salant, D. Insights into glomerular filtration and albuminuria. *N. Engl. J. Med.* **384**, 1437–1446 (2021). 33852781 10.1056/NEJMra1808786

[63] Eke, P. I. et al. Periodontitis in US Adults: National Health and Nutrition Examination Survey 2009-2014. *J. Am. Dent. Assoc.* **149**, 576–588.e576 (2018). 29957185 10.1016/j.adaj.2018.04.023 PMC8094373

[64] Jagodzinski, A. et al. Rationale and Design of the Hamburg City Health Study. *Eur. J. Epidemiol.* **35**, 169–181 (2020). 31705407 10.1007/s10654-019-00577-4 PMC7125064

[65] Organisation for Economic Co-operation and Development: Classifying educational programmes: manual for ISCED-97 implementation in OECD countries; 1999 edition. (1999).

[66] Boeing, H., Wahrendorf, J. & Becker, N. EPIC-Germany—A source for studies into diet and risk of chronic diseases. *Eur. Investig. Cancer Nutr. Ann. Nutr. Metab.* **43**, 195–204 (1999). 10.1159/000012786 10592368

[67] Petersen, E. L. et al. Multi-organ assessment in mainly non-hospitalized individuals after SARS-CoV-2 infection: The Hamburg City Health Study COVID programme. *Eur. Heart J.* **43**, 1124–1137 (2022). 34999762 10.1093/eurheartj/ehab914 PMC8755397

[68] Schmidt-Lauber, C. et al. Kidney outcome after mild to moderate COVID-19. *Nephrol. Dial. Transpl.* **38**, 2031–2040 (2023). 10.1093/ndt/gfad008 PMC10468748 36657383

[69] Walther, C. et al. Association between periodontitis and depression severity - A cross-sectional study of the older population in Hamburg. *Brain Behav. Immun. Health* **34**, 100689 (2023). 37822872 10.1016/j.bbih.2023.100689 PMC10562758

[70] Schmidt-Lauber, C. et al. Prevalence and characteristics of chronic kidney disease in the Hamburg City Health Study. *Nephrol. Dial. Transpl.* **40**, 1632–1634 (2025). 10.1093/ndt/gfaf075 PMC12315801 40307671

[71] Levey, A. S. et al. A new equation to estimate glomerular filtration rate. *Ann. Intern. Med.* **150**, 604–612 (2009). 19414839 10.7326/0003-4819-150-9-200905050-00006 PMC2763564

[72] Stevens, P. E. & Levin, A. & Kidney Disease: Improving Global Outcomes Chronic Kidney Disease Guideline Development Work Group, M. Evaluation and management of chronic kidney disease: synopsis of the kidney disease: improving global outcomes 2012 clinical practice guideline. *Ann. Intern Med.* **158**, 825–830 (2013). 23732715 10.7326/0003-4819-158-11-201306040-00007

[73] Fox, C. S. et al. Associations of kidney disease measures with mortality and end-stage renal disease in individuals with and without diabetes: a meta-analysis. *Lancet* **380**, 1662–1673 (2012). 23013602 10.1016/S0140-6736(12)61350-6 PMC3771350

[74] Arnlov, J. et al. Low-grade albuminuria and incidence of cardiovascular disease events in nonhypertensive and nondiabetic individuals: the Framingham Heart Study. *Circulation* **112**, 969–975 (2005). 16087792 10.1161/CIRCULATIONAHA.105.538132

[75] Holtfreter, B. et al. ACES: A new framework for the application of the 2018 periodontal status classification scheme to epidemiological survey data. *J. Clin. Periodontol.* **51**, 512–521 (2024). 38385950 10.1111/jcpe.13965

[76] Kinane, D. F., Stathopoulou, P. G. & Papapanou, P. N. Periodontal diseases. *Nat. Rev. Dis. Prim.* **3**, 17038 (2017). 28805207 10.1038/nrdp.2017.38

[77] CKD Work Group, K. KDIGO 2024 Clinical Practice Guideline for the Evaluation and Management of Chronic Kidney Disease. *Kidney Int.* **105**, S117-S314 (2024). 10.1016/j.kint.2023.10.018 38490803

[78] Genco, R. J. & Borgnakke, W. S. Risk factors for periodontal disease. *Periodontol. 2000* **62**, 59–94 (2013). 23574464 10.1111/j.1600-0757.2012.00457.x

[79] Tingley, D., Yamamoto, T., Hirose, K., Keele, L. & Imai, K. mediation: R Package for Causal Mediation Analysis. *J. Stat. Softw.* **59**, 1–38 (2014).26917999

