# Supplementary material for: Association of periodontitis with reduced kidney function and albuminuria in early chronic kidney disease: a population-based study
Source: Int J Oral Sci. 2026 Apr 6;18:33. doi: 10.1038/s41368-026-00435-6 (PMC13050795; doi:10.1038/s41368-026-00435-6)
Supplement: Supplementary file 1 — Supplementary information [file 41368_2026_435_MOESM1_ESM.docx]

**Table S1. Baseline characteristics stratified by eGFR categories.**

| **Characteristics** | **Overall** | **eGFR** | | **p-value** |
| --- | --- | --- | --- | --- |
|  | N = 5,636 | **< 60**, N = 427 (7.4%) | **≥ 60**, N = 5229 (93%) |  |
| **Sociodemographics** | | | | |
| Sex |  |  |  | 0.436 |
| Male | 2,816 (50%) | 216 (52%) | 2,600 (50%) |  |
| Female | 2,820 (50%) | 201 (48%) | 2,619 (50%) |  |
| Age | 62.0 (55.0,69.0) | 71.0 (66.0,74.0) | 62.0 (54.0,68.0) | <0.001 |
| Education |  |  |  | <0.001 |
| Low | 236 (4.4%) | 27 (6.8%) | 209 (4.2%) |  |
| Medium | 2,662 (49%) | 231 (59%) | 2,431 (49%) |  |
| High | 2,482 (46%) | 134 (34%) | 2,348 (47%) |  |
| (Missing) | 256 | 25 | 231 |  |
| BMI | 25.9 (23.5,28.9) | 28.0 (25.2,31.5) | 25.8 (23.4,28.7) | <0.001 |
| (Missing) | 283 | 29 | 254 |  |
| **Risk factors** | | | | |
| Smoking |  |  |  | 0.035 |
| Never | 2,074 (37%) | 152 (36%) | 1,922 (37%) |  |
| Current | 1,029 (18%) | 57 (14%) | 962 (19%) |  |
| Former | 2,516 (45%) | 204 (49%) | 2,312 (44%) |  |
| (Missing) | 27 | 4 | 23 |  |
| Alcohol g/day | 9.7 (2.8,23.2) | 4.6 (1.2,14.9) | 10.1 (3.0,23.5) | <0.001 |
| (Missing) | 494 | 61 | 433 |  |
| Diabetes | 399 (7.5%) | 72 (18%) | 327 (6.6%) | <0.001 |
| (Missing) | 289 | 18 | 271 |  |
| Hypertension | 3,470 (64%) | 363 (89%) | 3,107 (62%) | <0.001 |
| (Missing) | 244 | 7 | 237 |  |
| Dyslipidemia | 1,246 (23%) | 168 (42%) | 1,078 (22%) | <0.001 |
| (Missing) | 255 | 14 | 241 |  |
| Coronary artery disease | 205 (3.9%) | 38 (10%) | 167 (3.4%) | <0.001 |
| (Missing) | 371 | 46 | 325 |  |
| **Inflammatory biomarkers** | | | | |
| IL-6 (pg/ml) | 1.57 (1.14,2.20) | 2.22 (1.67,3.19) | 1.53 (1.12,2.13) | <0.001 |
| (Missing) | 2,537 | 225 | 2,312 |  |
| hsCRP (mg/dl) | 0.12 (0.06,0.25) | 0.19 (0.09,0.43) | 0.11 (0.06,0.24) | <0.001 |
| (Missing) | 184 | 10 | 174 |  |
| **Kidney function** | | | | |
| uACR (mg/g) | 4.2 (2.5,8.0) | 6.5 (3.1,15.3) | 4.1 (2.4,7.5) | <0.001 |
| (Missing) | 854 | 57 | 797 |  |
| eGFR (ml/min/1.73m^2^) | 81.5 (71.9,91.2) | 54.0 (47.8,57.1) | 82.9 (74.3,91.9) | <0.001 |
| **Dental parameters** | | | | |
| Periodontitis |  |  |  | <0.001 |
| Stage I/II | 2,054 (36%) | 94 (23%) | 1,960 (38%) |  |
| Stage III | 2,374 (42%) | 172 (41%) | 2,202 (42%) |  |
| Stage IV | 1,208 (21%) | 151 (36%) | 1,057 (20%) |  |
|  |  |  |  |  |
| Sites/mouth CAL ≥ 3mm | 36.4 (19.8,57.4) | 45.8 (28.3,67.3) | 35.8 (19.1,56.7) | <0.001 |
| Mean CAL | 2.4 (2.0,2.8) | 2.6 (2.2,3.2) | 2.4 (2.0,2.8) | <0.001 |
| Cumulative CAL > 3mm | 61.0 (21.0,136.5) | 88.0 (37.0,174.0) | 59.0 (20.0,133.0) | <0.001 |
|  |  |  |  |  |
|  |  |  |  |  |
| Sites/mouth PD ≥ 4mm | 2.4 (0.0,8.0) | 4.8 (1.2,12.0) | 2.1 (0.0,7.6) | <0.001 |
| Mean PD | 2.1 (1.8,2.4) | 2.2 (1.9,2.6) | 2.1 (1.8,2.4) | <0.001 |
|  |  |  |  |  |
| Plaque Index | 7.7 (0.0,27.3) | 13.1 (0.0,42.3) | 7.4 (0.0,26.8) | <0.001 |
| (Missing) | 75 | 7 | 68 |  |
| DMFT-Index | 19.0 (15.0,23.0) | 22.0 (18.0,25.0) | 19.0 (15.0,22.0) | <0.001 |
| BOP Index | 7.7 (1.9,20.4) | 9.8 (3.3,23.1) | 7.4 (1.9,20.0) | 0.002 |
| (Missing) | 93 | 7 | 86 |  |
|  |  |  |  |  |
|  |  |  |  |  |
| PISA | 80.8 (18.3,234.3) | 111.5 (26.5,251.7) | 78.2 (17.8,232.9) | 0.008 |
| (Missing) | 93 | 7 | 86 |  |
| Number of missing teeth | 2.0 (1.0,5.0) | 4.0 (2.0,9.0) | 2.0 (1.0,5.0) | <0.001 |
|  |  |  |  |  |

Numbers are median (IQR) for continuous and n (%) for categorical parameters. Pearson’s chi-squared, Wilcoxon rank sum, or Fisher’s exact test were used for comparison between eGFR categories. CAL: clinical attachment loss, BMI: body mass index, BOP: bleeding on probe, eGFR: estimated glomerular filtration rate, hsCRP: high-sensitive C-reactive protein, IL-6: interleukin 6, PD: probing depth, uACR: urinary albumin-to-creatinine ratio.

**Table S2. Baseline characteristics stratified by uACR stages.**

| **Characteristics** | **Overall** | **uACR** | | **p-value** |
| --- | --- | --- | --- | --- |
|  | N = 5,229 | **< 30**, N = 4913 (94%) | **≥ 30**, N = 316 (6.0%) |  |
| **Sociodemographics** | | | | |
| Sex |  |  |  | <0.001 |
| Male | 2,597 (50%) | 2,404 (49%) | 193 (61%) |  |
| Female | 2,632 (50%) | 2,509 (51%) | 123 (39%) |  |
| Age | 63.0 (55.0,70.0) | 62.0 (55.0,69.0) | 68.0 (60.0,72.0) | <0.001 |
| Education |  |  |  | 0.097 |
| Low | 231 (4.6%) | 217 (4.6%) | 14 (4.8%) |  |
| Medium | 2,444 (49%) | 2,283 (49%) | 161 (55%) |  |
| High | 2,307 (46%) | 2,189 (47%) | 118 (40%) |  |
| (Missing) | 247 | 224 | 23 |  |
| BMI | 26.1 (23.6,29.0) | 26.0 (23.5,28.9) | 27.5 (24.3,31.1) | <0.001 |
| (Missing) | 263 | 243 | 20 |  |
| **Risk factors** | | | | |
| Smoking |  |  |  | 0.582 |
| Never | 1,882 (36%) | 1,774 (36%) | 108 (35%) |  |
| Current | 955 (18%) | 891 (18%) | 64 (21%) |  |
| Former | 2,362 (45%) | 2,222 (45%) | 140 (45%) |  |
| (Missing) | 30 | 26 | 4 |  |
| Alcohol g/day | 9.7 (2.8,23.1) | 9.8 (2.8,23.1) | 8.3 (1.9,22.0) | 0.115 |
| (Missing) | 482 | 436 | 46 |  |
| Diabetes | 384 (7.9%) | 317 (6.9%) | 67 (23%) | <0.001 |
| (Missing) | 370 | 347 | 23 |  |
| Hypertension | 3,253 (65%) | 2,991 (64%) | 262 (85%) | <0.001 |
| (Missing) | 218 | 210 | 8 |  |
| Dyslipidemia | 1,189 (24%) | 1,079 (23%) | 110 (38%) | <0.001 |
| (Missing) | 334 | 309 | 25 |  |
| Coronary artery disease | 199 (4.1%) | 180 (3.9%) | 19 (7.0%) | 0.013 |
| (Missing) | 362 | 318 | 44 |  |
| **Inflammatory biomarkers** | | | | |
| IL-6 (pg/ml) | 1.57 (1.15,2.22) | 1.56 (1.14,2.17) | 1.96 (1.46,2.75) | <0.001 |
| (Missing) | 2,688 | 2,517 | 171 |  |
| hsCRP (mg/dl) | 0.12 (0.06,0.25) | 0.11 (0.06,0.24) | 0.16 (0.07,0.39) | <0.001 |
| (Missing) | 298 | 279 | 19 |  |
| **Kidney function** | | | | |
| uACR (mg/g) | 4.2 (2.5,8.1) | 3.9 (2.4,7.0) | 63.0 (41.0,132) | <0.001 |
| eGFR (ml/min/1.73m^2^) | 81.1 (71.6,90.9) | 81.4 (72.2,91.1) | 73.1 (61.8,86.1) | <0.001 |
| (Missing) | 447 | 405 | 42 |  |
| **Dental parameters** | | | | |
| Periodontitis |  |  |  | <0.001 |
| Stage I/II | 1,887 (36%) | 1,798 (37%) | 89 (28%) |  |
| Stage III | 2,187 (42%) | 2,063 (42%) | 124 (39%) |  |
| Stage IV | 1,155 (22%) | 1,052 (21%) | 103 (33%) |  |
|  |  |  |  |  |
|  |  |  |  |  |
| Sites/mouth CAL ≥ 3mm | 37.2 (20.0,57.7) | 36.7 (19.8,57.4) | 46.4 (27.1,64.7) | <0.001 |
| Mean CAL | 2.4 (2.0,2.9) | 2.4 (2.0,2.8) | 2.6 (2.2,3.1) | <0.001 |
| Cumulative CAL > 3mm | 61.0 (21.0,137.0) | 60.0 (21.0,135.0) | 77.0 (36.5,170.5) | <0.001 |
|  |  |  |  |  |
| Sites/mouth PD ≥ 4mm | 2.4 (0.0,8.0) | 2.4 (0.0,8.0) | 4.2 (0.8,10.7) | <0.001 |
| Mean PD | 2.1 (1.8,2.4) | 2.1 (1.8,2.4) | 2.2 (1.9,2.5) | <0.001 |
|  |  |  |  |  |
|  |  |  |  |  |
| Plaque Index | 8.0 (0.0,27.3) | 7.7 (0.0,26.9) | 14.1 (0.0,37.5) | 0.002 |
| (Missing) | 74 | 67 | 7 |  |
|  |  |  |  |  |
|  |  |  |  |  |
| DMFT-Index | 19.0 (16.0,23.0) | 19.0 (15.0,23.0) | 21.0 (17.0,24.5) | <0.001 |
| BOP Index | 7.7 (2.0,20.4) | 7.7 (2.0,20.4) | 8.9 (1.9,21.2) | 0.845 |
| (Missing) | 86 | 82 | 4 |  |
| PISA | 82.2 (19.3,233.7) | 81.4 (19.4,233.5) | 88.0 (15.8,234.9) | 0.900 |
| Unknown | 86 | 82 | 4 |  |
| Number of missing teeth | 2.0 (1.0,6.0) | 2.0 (1.0,5.0) | 4.0 (1.0,8.0) | <0.001 |

Numbers are median (IQR) for continuous and n (%) for categorical parameters. Pearson’s chi-squared, Wilcoxon rank sum, or Fisher’s exact test were used for comparison between uACR categories. CAL: clinical attachment loss, BMI: body mass index, BOP: bleeding on probe, eGFR: estimated glomerular filtration rate, hsCRP: high-sensitive C-reactive protein, IL-6: interleukin 6, PD: probing depth, uACR: urinary albumin-to-creatinine ratio.

**Table S3. Interaction analysis of dental parameters with demographic and clinical covariates in relation to and kidney function parameters.**

**a)**

|  |  | | **Stage III versus Stage I/II** | |  | **Stage IV versus Stage I/II** | |  |
| --- | --- | --- | --- | --- | --- | --- | --- | --- |
| **Outcome** | **Interaction** | **Estimate**  **(95%-CI)** | | **p- value**  **for interaction** |  | **Estimate**  **(95%-CI)** | **p- value**  **for interaction** | |
| eGFR | age (≥ 60) | 0.29 (-1.31 – 1.89) | | 0.724 |  | 0.15 (-2.06 – 2.36) | 0.892 | |
| eGFR | Sex (female) | 0.4 (-1.13 – 1.92) | | 0.61 |  | 1.65 (-0.17 – 3.48) | 0.076 | |
| eGFR | Smoking (yes) | -0.57 (-2.63 – 1.49) | | 0.587 |  | 0.62 (-1.61 – 2.85) | 0.588 | |
| eGFR | Diabetes (yes) | 1.12 (-2.04 – 4.28) | | 0.487 |  | -1.96 (-5.28 – 1.35) | 0.246 | |
| uACR | Age ≥ 60 | -0.00 (-0.13 – 0.12) | | 0.942 |  | 0.06 (-0.11 – 0.23) | 0.493 | |
| uACR | Sex (female) | -0.08 (-0.21 – 0.04) | | 0.175 |  | -0.19 (-0.33 – -0.04) | 0.012 | |
| uACR | Smoking (yes) | -0.14 (-0.30 – 0.03) | | 0.107 |  | -0.13 (-0.30 – 0.05) | 0.163 | |
| uACR | Diabetes (yes) | 0.00 (-0.24 – 0.25) | | 0.971 |  | 0.19 (-0.06 – 0.45) | 0.14 | |

**b)**

| **Outcome** | **Interaction** | **Estimate**  **(95%-CI)** | **p- value**  **for interaction** |  |
| --- | --- | --- | --- | --- |
| eGFR | Age (≥ 60) | -0.45 (-1.47 – 0.56) | 0.384 |  |
| eGFR | Sex (female) | 0.19 (-0.73 – 1.12) | 0.685 |  |
| eGFR | Smoking (yes) | -0.01 (-0.98 – 0.95) | 0.976 |  |
| eGFR | Diabetes (yes) | -1.37 (-2.79 – 0.06) | 0.06 |  |
| uACR | Age (≥ 60) | 0.02 (-0.05 – 0.09) | 0.6 |  |
| uACR | Sex (female) | -0.05 (-0.12 – 0.02) | 0.155 |  |
| uACR | Smoking (yes) | 0.1 (-0.1 – 0.3) | 0.319 |  |
| uACR | Diabetes (yes) | 0.08 (-0.02 – 0.18) | 0.114 |  |

The table shows interaction terms of a) periodontitis staged according to the 2017 AAP/EFP periodontitis definition with Stage I/II as reference category and b) mean CAL with eGFR or uACR as indicated. Fully adjusted models include age, sex, diabetes, and smoking. For interaction analyses, age was categorized into < or ≥ 60 years. CAL: clinical attachment loss, CI: confidence interval, eGFR: estimated glomerular filtration rate, uACR: urinary albumin-to-creatinine ratio.

**Table S4. Generalized variance inflation factors (GVIF) for adjusted linear regression models.**

**a)**

|  | **eGFR** | | |  | **uACR** | | |
| --- | --- | --- | --- | --- | --- | --- | --- |
| **Variable** | **GVIF** | **DF** | **scaled GVIF** |  | **GVIF** | **DF** | **scaled GVIF** |
| Periodontitis | 1.131 | 2 | 1.031 |  | 1.127 | 2 | 1.030 |
| Age | 1.134 | 1 | 1.065 |  | 1.131 | 1 | 1.063 |
| Sex | 1.032 | 1 | 1.016 |  | 1.033 | 1 | 1.016 |
| Diabetes | 1.023 | 1 | 1.012 |  | 1.025 | 1 | 1.012 |
| Smoking | 1.073 | 2 | 1.018 |  | 1.073 | 2 | 1.018 |

**b)**

|  | **eGFR** | | |  | **uACR** | | |
| --- | --- | --- | --- | --- | --- | --- | --- |
| **Variable** | **GVIF** | **DF** | **scaled GVIF** |  | **GVIF** | **DF** | **scaled GVIF** |
| Mean CAL | 1.102 | 1 | 1.050 |  | 1.103 | 1 | 1.050 |
| Age | 1.091 | 1 | 1.044 |  | 1.091 | 1 | 1.045 |
| Sex | 1.040 | 1 | 1.020 |  | 1.041 | 1 | 1.020 |
| Diabetes | 1.023 | 1 | 1.011 |  | 1.025 | 1 | 1.012 |
| Smoking | 1.078 | 2 | 1.019 |  | 1.081 | 2 | 1.020 |

The table shows the GVIF of adjusted linear regression models for a) periodontitis stages according to the 2017 AAP/EFP periodontitis definition (Stage IV versus Stage I/II as reference category) and b) mean CAL on the eGFR or uACR as indicated. CAL: clinical attachment loss, eGFR: estimated glomerular filtration rate, GVIF: generalized variance inflation factors, uACR: urinary albumin-to-creatinine ratio.

**Figure S1. Mediation of IL-6 on the association between mean CAL and kidney function.**


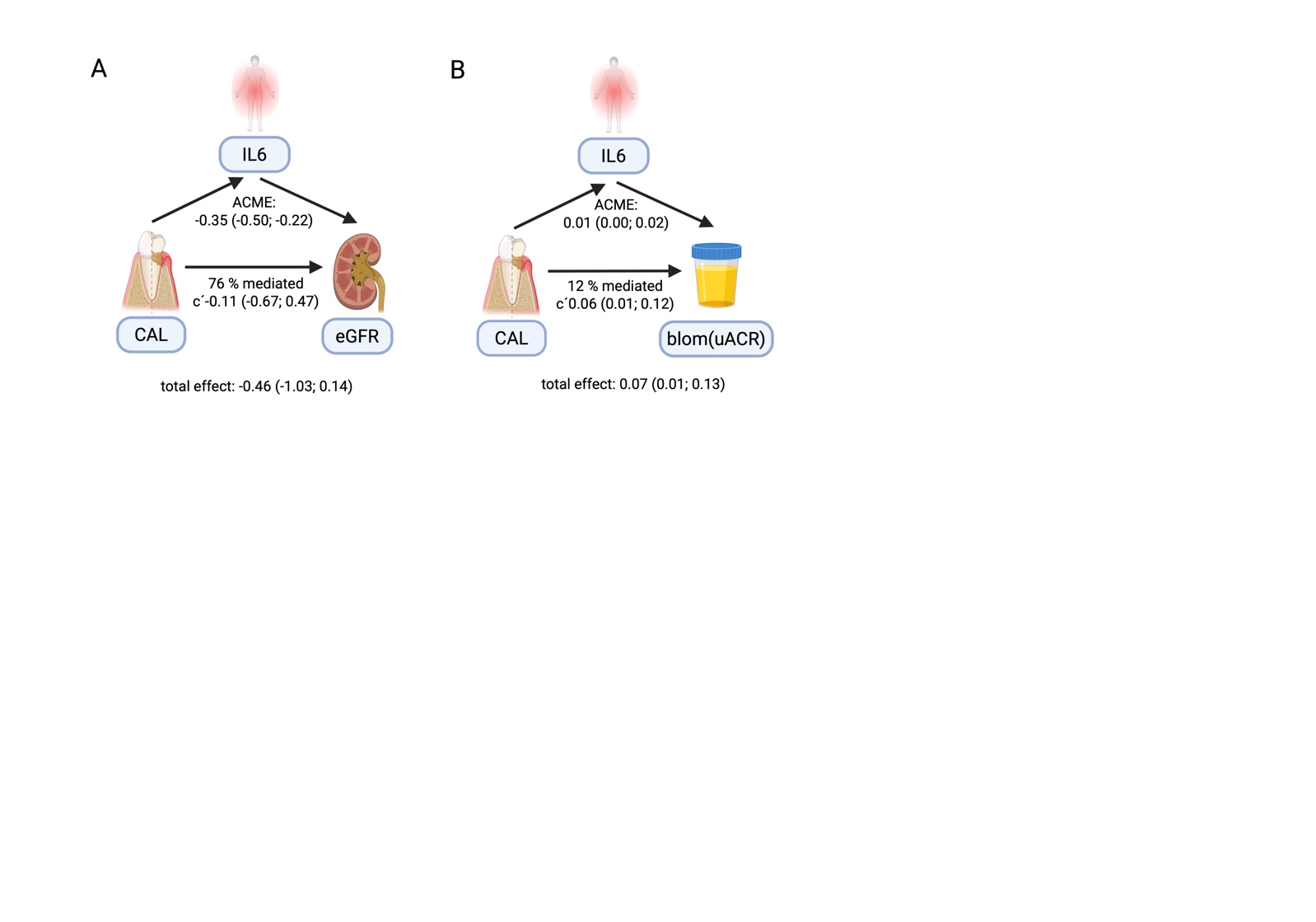


Graphic shows results from causal mediation analyses investigating the mediating effect of the inflammatory marker IL-6 on the association between mean CAL and eGFR (A) or the Blom-transformed uACR (B) with adjustment for age, sex, diabetes, and smoking status. ACME: average causal mediation effect, CAL: clinical attachment loss, eGFR: estimated glomerular filtration rate, IL-6: interleukin 6, uACR: urine albumin-to-creatinine ratio. Created in BioRender. Schmidt-Lauber, C. (2026) https://BioRender.com/do5pdzd.

**Figure S2. Mediation of IL-6 on the association between periodontitis and kidney function.**


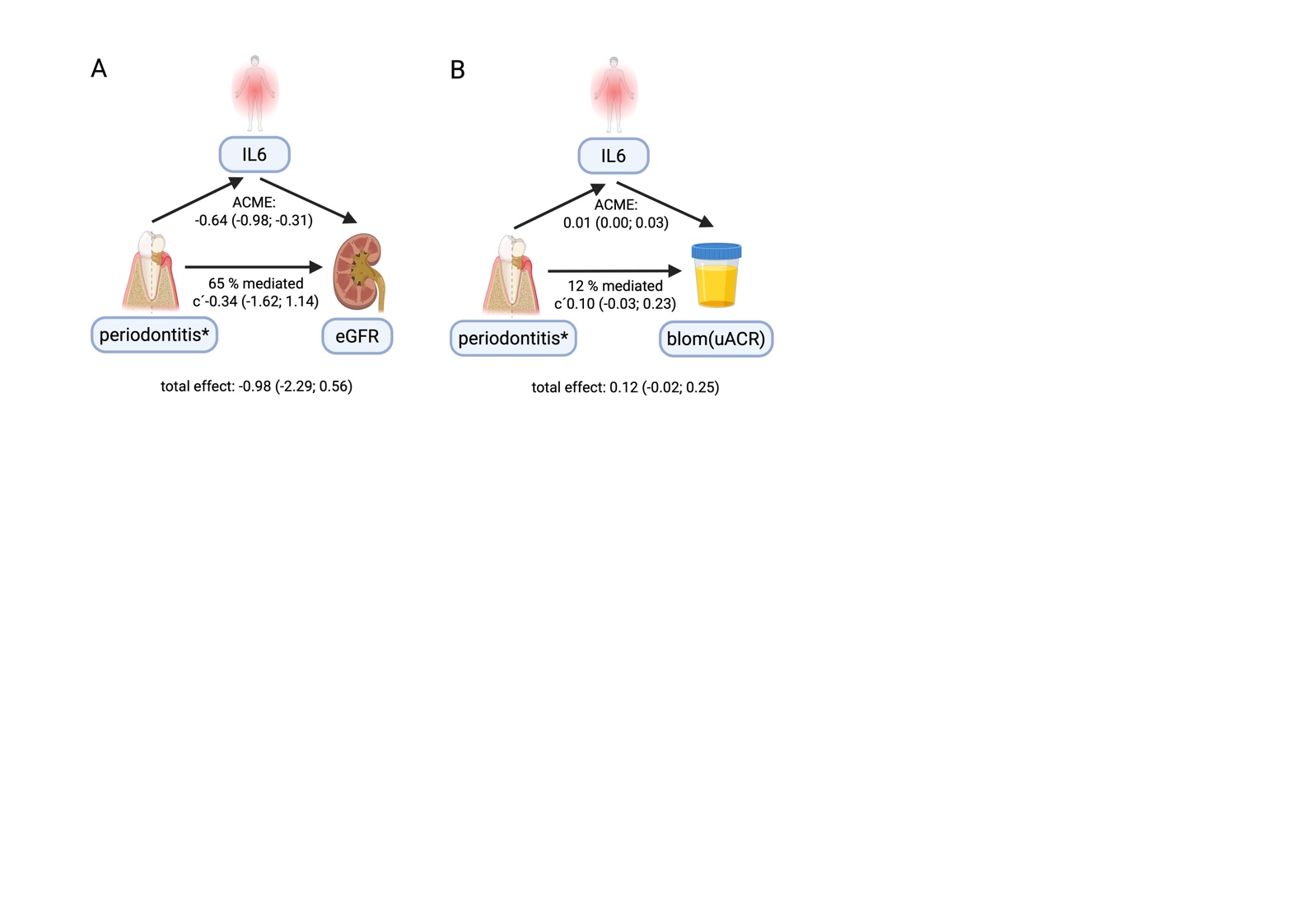


Graphic shows results from causal mediation analyses investigating the mediating effect of the inflammatory marker IL-6 on the association between *Stage I/II vs Stage IV periodontitis and eGFR (A) or the Blom-transformed uACR (B) with adjustment for age, sex, diabetes, and smoking status. ACME: average causal mediation effect, eGFR: estimated glomerular filtration rate, IL-6: interleukin 6, uACR: urine albumin-to-creatinine ratio. Created in BioRender. Schmidt-Lauber, C. (2026) https://BioRender.com/fz2y5tn.
